# Positive Psychological Well-Being and Determinants of Social Robot Acceptability Among Patients With Heart Failure: Cross-Sectional Questionnaire Study

**DOI:** 10.2196/83163

**Published:** 2026-06-02

**Authors:** Lisa-Marie Maukel, Karen Bouchard, Jess G Fiedorowicz, Kerstin Dautenhahn, Moojan Ghafurian, Thais Coutinho, Caroline McGuinty, Peter P Liu, Heather Tulloch

**Affiliations:** 1 Division of Prevention and Rehabilitation, University of Ottawa Heart Institute, 40 Ruskin Street, l, Ottawa, ON, K1Y4W7, Canada, 1 6132234340; 2 Faculty of Medicine, University of Ottawa, Ottawa, ON, Canada; 3 Department of Mental Health, The Ottawa Hospital, Ottawa, ON, Canada; 4 Department Systems Design Engineering, University of Waterloo, Waterloo, ON, Canada; 5 Department of Cardiovascular Medicine, Mayo Clinic, Rochester, MN, United States; 6 Division of Cardiology, University of Ottawa Heart Institute, Ottawa, ON, Canada

**Keywords:** heart failure, social robots, robotics, technology acceptability, positive psychological well-being, assistive technology, mental health

## Abstract

**Background:**

Social robots (SRs) are innovative tools in health care, offering both medical and psychological support for patients with heart failure (HF). For successful implementation, patient acceptability of SRs is crucial. Living in urban areas and having a lower comorbidity burden have been linked to higher acceptability; however, the role of psychological factors remains underexplored.

**Objective:**

This study aimed to examine the associations between negative (eg, depression and anxiety) and positive (eg, optimism) psychological factors and personality traits (eg, openness and extraversion) with SR acceptability in patients with HF.

**Methods:**

Patients with HF watched brief videos about SRs and completed validated measures of depressive symptoms (Patient Health Questionnaire-9), anxiety symptoms (Generalized Anxiety Disorder-7), positive psychological well-being (Brief Inventory of Thriving), and personality traits (Ten-Item Personality Inventory). Medical information was extracted from patients’ records. SR acceptability was assessed using the Unified Theory of Acceptance and Use of Technology (UTAUT). Pearson correlations and multiple linear regression, adjusted for age, sex, smart technology experience, urbanicity, and comorbidities, were conducted.

**Results:**

Of the 101 patients (women: n=36, 35.6%, mean age 68, SD 10 y), 23% (23/101) scored in the clinical range for depression, and 17% (17/101) scored in the clinical range for anxiety. Well-being scores were moderate, and conscientiousness and agreeableness were the most common. UTAUT behavioral intention was moderate; 69% (67/97) of participants were likely to use an SR if available. Well-being scores correlated positively with SR acceptability in 4 of 5 UTAUT subscales, whereas no significant bivariate associations were observed for psychological distress or personality traits. In the multiple regression models, higher Brief Inventory of Thriving scores were associated with increased SR acceptability, including UTAUT facilitating conditions (*B*=0.17; *P*=.01) and behavioral intention (*B*=0.17; *P*=.04), independent of depressive and anxiety symptoms.

**Conclusions:**

Psychological well-being is associated with determinants of SR acceptability in patients with HF, while psychological distress and personality traits are not associated with these determinants. These patient-level factors ought to be examined more closely before SR implementation.

## Introduction

Heart failure (HF) is a growing global health concern, affecting approximately 56.2 million people worldwide [1]. The condition is characterized by fluctuating functional impairment, frequent exacerbations, and high hospitalization rates, with nearly 50% of patients with HF being readmitted after an initial hospital stay [2]. These recurrent hospitalizations lead to a significant decline in quality of life and contribute to a 5-year mortality rate of 50% [3,4]. Moreover, HF imposes substantial economic burdens, with annual health care costs in the United States reaching US $30.7 billion [5]. The unpredictable trajectory, severe symptom burden of HF [4], and the high clinical demand [6] highlight the need for innovative solutions to improve patient support and disease management, and reduce resource burden.

One emerging technological advancement is the use of social robots (SRs) [7]. These artificial intelligence–powered agents, designed to mimic human or animal characteristics, engage users through verbal, nonverbal, and affective interactions [8]. SRs can assist in a variety of areas, including health monitoring (eg, blood pressure tracking), daily self-care tasks (eg, medication adherence), rehabilitation, and providing companionship [8-10]. By simulating health care provider interactions and facilitating home-based care [11], SRs align with the increasing demand for strategies that reduce hospital readmissions and improve patient satisfaction, particularly for those with limited access to health care services [12].

Research in older adults has demonstrated that SRs can positively impact physiological markers, such as pulse rate, cortisol levels, blood pressure, and oxygenation [13]. Additionally, SRs have been shown to promote exercise and medication adherence, reduce unhealthy snacking, and support weight loss [14-18]. Among those with dementia, SRs have effectively addressed psychosocial needs by alleviating loneliness and depressive symptoms [19-21], factors particularly relevant for patients with HF [22].

Despite these promising findings, the application of SRs in cardiac care remains limited. Before implementing novel technology, assessing acceptability within the target population is crucial [11,23,24]. In contrast to acceptance, which refers to the actual adoption or sustained use of a technology, acceptability reflects an individual’s behavioral intention to use a technology, influenced by factors such as performance expectations, usability, privacy, and patient characteristics [25-27]. So far, only one small study (n=3) examined SR use in patients with cardiovascular conditions, suggesting that SRs may improve exercise adherence during cardiac rehabilitation compared with usual care [28]. An accompanied survey of 28 patients with cardiac conditions revealed that 75% responded positively to SRs following direct interaction [29]. Our prior research involving HF health care providers, including cardiologists and cardiac nurses, indicated a general openness to using SRs for data collection and patient prompts [30]. Similarly, our study including patients with HF found that 69% would be willing to use an SR if available, with higher acceptability observed among those living in urban areas and experiencing a lower symptom burden [31]. These findings offer initial insights into potential determinants of SR acceptability in the HF population.

Psychological factors likely play a key role in shaping SR acceptability. Depressive symptoms are associated with reduced approach motivation and behavioral withdrawal, whereas anxiety increases avoidance and threat sensitivity, particularly in contexts involving uncertainty [32]. Within this framework, depressive and anxious symptoms may act as barriers to SR acceptability. However, empirical findings are inconsistent: some studies report higher SR acceptability with negative affect [33], while another study found no such connection with depressive mood [34]. Although technology-specific anxiety consistently predicts lower SR acceptability [24,27,35], the influence of generalized anxiety remains unclear, both in healthy individuals and in clinical populations. One experimental study found that fear decreased the likelihood of interacting with SRs in a student sample [36], but its relevance to HF populations remains unclear. Thus, further investigation into the relationship between anxiety and depression with SR acceptability is warranted.

In contrast, positive psychological factors, such as positive psychological well-being (PPWB), activate approach-oriented systems that promote exploration, goal-directed behavior, and openness to novelty [32,37]. In the context of SRs, positive emotions, such as enjoyment and excitement, may increase acceptability of SRs [36,38], potentially by reducing perceived effort and fostering engagement [39].

PPWB, including optimism, purpose, resilience, gratitude, positive affect, and happiness, is increasingly recognized as a critical factor in HF care [40-42]. The 2021 American Heart Association scientific statement on *psychological health, well-being, and the mind-heart-body connection* underscores the association of PPWB with reduced cardiovascular disease risk and lower mortality rates [43]. Importantly, PPWB represents a construct distinct from the mere absence of depression or negative affect and is independently associated with health outcomes [44,45]. However, its relevance to SR acceptability in cardiac populations has not yet been examined.

Beyond affective states, personality traits may also influence SR acceptability. The Five-Factor Model (Big Five) is the most widely recognized framework for describing individual differences in personality [46], comprising neuroticism, extraversion, agreeableness, conscientiousness, and openness to experience [47]. Within an approach-avoidance framework, these traits may shape how individuals evaluate and engage with SRs. Extraversion, characterized by social engagement and reward sensitivity, aligns with approach tendencies and may be associated with more positive attitudes toward SRs. In contrast, neuroticism, marked by threat sensitivity and negative affect, aligns with avoidance tendencies and may reduce acceptability [48]. Openness to experience may enhance receptivity to novel technologies, while conscientiousness and agreeableness may foster engagement through perceived usefulness and trust in the SR as a supportive tool.

These traits have already been shown to be essential for understanding health behaviors and cardiovascular outcomes [49]. Specifically, agreeableness and conscientiousness have been linked to better adherence to treatment regimens [50], which could also apply to SR interventions delivered by health care providers. Although traits such as agreeableness, extraversion, and openness have been found to influence SR acceptability in general populations [51,52], their impact on patients with HF remains unknown.

Understanding the relationship between these psychological factors and SR acceptability in patients with HF is crucial for developing SR interventions that cater to patients’ specific needs and preferences, potentially improving engagement and overall treatment outcomes. As such, this study aimed to examine the relationships between negative (depression and anxiety) and positive (PPWB) psychological factors, personality traits, and SR acceptability in patients with HF.

## Methods

### Study Population and Recruitment

Patients with HF were recruited from the University of Ottawa Heart Institute, a large tertiary and quaternary cardiac care center serving a population of 1.4 million in the Champlain Local Health Integration Network, Ontario, Canada. Outpatient participants were enrolled between September 2022 and December 2023. Eligible participants had a diagnosis of either HF with preserved ejection fraction (left ventricular ejection fraction [LVEF] ≥50%) or HF with reduced ejection fraction (HFrEF; LVEF <40%) and were symptomatic, as defined by New York Heart Association (NYHA) functional class II-IV, indicating at least mild limitations in physical activity [53]. Additional inclusion criteria included being over 18 years of age, proficient in reading either English or French, and capable of providing informed consent. Exclusion criteria included individuals with unstable psychiatric conditions (eg, suicidal ideation or unmanaged psychotic disorders) or severe cognitive impairment requiring substantial assistance with daily activities.

Participants were recruited through clinician referrals and a review of medical records. Potentially eligible participants were contacted either via phone or in person at the clinic. A research coordinator confirmed eligibility and obtained informed consent. To reduce selection bias, we attempted to approach all eligible patients during the study period. Participants had the option to complete the survey using a secure web-based platform, REDCap (Research Electronic Data Capture) [54], or on paper, either on-site or at home. Before completing the study questionnaires, all patients were shown 4 brief informational manufacturer videos of SR models (Mabu, Catalia Health Inc.; PARO, Paro Therapeutic Robot; Elli-Q, Intuition Robotics; and Pepper, SoftBank Robotics), following established methods to introduce participants to new technologies [55]. The videos demonstrated SR functions such as scheduling appointments, reminding patients about daily exercises and medications, and offering companionship and social connection. Full descriptions and transcripts of all videos are provided in Supplement 1 in Multimedia Appendix 1. For participants without access to the necessary technology, the videos were presented on a tablet during their clinic visit.

### Ethical Considerations

This study received approval from the Ottawa Health Science Network Research Ethics Board (Protocol ID: 20210165‐01H). Written informed consent was obtained from all participants. All study data were deidentified prior to analysis to protect participant privacy. Only study IDs were used, and no personal identifiers were linked to the dataset. Participants received no compensation.

### Measurements

#### Demographic and Medical Information

Participants completed a questionnaire of demographic characteristics (eg, sex, urbanicity, and ethnicity) and medical conditions. These data were verified by their hospital records. Urbanicity was dichotomized to compare urban versus nonurban residence (1=urban; and 0=suburban, mixed, or rural). The Charlson Comorbidity Index (CCI), a weighted tool that quantifies comorbid conditions by accounting for both their number and severity, was calculated to assess the comorbidity burden. Scores range from 0 to 37, with higher scores indicating a greater burden of comorbid diseases [56].

#### General Attitudes Toward Technology and SRs

On the basis of previous research on SR acceptability in older adults [23], participants completed an 11-item questionnaire assessing their general attitudes toward and experiences with technology and SRs. Example items included “Have you interacted with a social robot in the past?*”* (yes or no) and “I would use a social robot if it were to become available to me*”* (agree, somewhat agree, somewhat disagree, or disagree) dichotomized to “Would likely use SR” (yes or no). To capture prior experience with smart technology, we calculated a sum score based on the question *“*Please select what type of smart devices do you currently have?” Options included smartphone, wearable devices, voice assistant agents, and robotic vacuum cleaners, resulting in a possible range of 0 to 12 smart devices in use. In addition, patients ranked the perceived usefulness of various SR capabilities and features using a 25-item questionnaire [10,23], for providing in-home care. Each item was rated on a 5-point Likert scale, ranging from 1 (extremely useless) to 5 (extremely useful). Participants evaluated capabilities such as management and communication (eg, scheduling appointments), monitoring (eg, tracking blood pressure), medical procedures and treatment (eg, administering oral medications), emotional support (eg, providing companionship), and daily living assistance (eg, assisting with eating). This questionnaire is provided in Supplement 2 in Multimedia Appendix 1.

#### Depressive Symptoms

Depressive symptoms were evaluated using the Patient Health Questionnaire-9 (PHQ-9) [57,58], a well-established instrument frequently applied with cardiovascular populations [59]. Participants rated 9 items on a 4-point Likert scale, ranging from 0 (not at all) to 3 (nearly every day), generating a total score range from 0 to 27, with higher scores reflecting higher symptom severity. A score of 10 or higher was used as a cutoff for moderate depressive symptoms [57,60]. The tool exhibits strong psychometric properties, including good internal consistency (Cronbach α=0.89) and robust validity [57]. The PHQ-9 demonstrated good internal consistency in this sample (Cronbach α=0.86).

#### Generalized Anxiety

Anxiety symptoms were assessed using the Generalized Anxiety Disorder-7 (GAD-7) scale [61]. Participants rated 7 items on a 4-point Likert scale, ranging from 0 (not at all) to 3 (nearly every day), with total scores ranging from 0 to 21. Higher scores indicate greater anxiety symptom severity. A score of 10 or higher is considered the optimal threshold for screening generalized anxiety. In a cardiac sample, the GAD-7 demonstrated strong reliability, with a Cronbach α of 0.89 and a composite reliability index of 0.90. It also exhibited robust validity across procedural, criterion, construct, and factorial measures [62]. The GAD-7 demonstrated excellent internal consistency in this sample (Cronbach α=0.93).

#### Positive Psychological Well-Being

PPWB was assessed using the Brief Inventory of Thriving (BIT) [63], a 10-item scale measuring positive functioning across multiple well-being domains, including life satisfaction, positive emotions, supportive relationships, engagement, purpose, mastery, and optimism. Each item was scored on a 5-point scale, ranging from 1 (strongly disagree) to 5 (strongly agree), with higher scores representing greater well-being. Total scores ranged from 10 to 50. Sample items included “There are people who appreciate me as a person” and “What I do in life is valuable and worthwhile.*”* The BIT has been shown to have strong psychometric properties, including retest reliability and convergent validity [63]. The BIT demonstrated excellent internal consistency in this HF sample (Cronbach α=0.92).

#### Personality Style

The Ten-Item Personality Inventory (TIPI) [64] was used to assess the 5-factor model of personality: extraversion, agreeableness, conscientiousness, emotional stability (neuroticism), and openness to experience. Two items representing each trait are rated on a 7-point Likert scale, ranging from 1 (disagree strongly) to 7 (agree strongly). Higher scores reflected stronger tendencies toward the respective traits. The TIPI demonstrated acceptable test-retest reliability, structural validity, and convergent validity [65]. The TIPI has been applied before in cardiac samples [66]. In this HF sample, Cronbach α was 0.40 for extraversion, 0.33 for agreeableness, 0.33 for conscientiousness, 0.66 for emotional stability, and 0.49 for openness. These modest values are consistent with expectations for 2-item TIPI subscales [64].

#### Acceptability of SRs

To assess patients’ acceptability of SRs, we used the 19-item Unified Theory of Acceptance and Use of Technology (UTAUT) questionnaire [67,68] adapted for SRs. Each item was rated on a 7-point Likert scale, ranging from 1 (strongly disagree) to 7 (strongly agree). The UTAUT evaluates 5 key factors that influence the behavioral intention to use SRs, with subscale scores ranging from 4 to 28. Higher scores on each subscale indicate greater acceptability and a higher likelihood of SR usage. The factors assessed include performance expectancy (eg, *“*I would find a social robot useful in my everyday life*”*), effort expectancy (eg, “I would find the social robot easy to use*”*), facilitating conditions (eg, “I would have the resources necessary to use a social robot*”*), and social influence (eg, “People who are important to me think that I should use a social robot*”*). Behavioral intention to use an SR, with a score range of 3 to 21, was measured with items such as “If it was available to me, I intend to use a social robot.*”* The UTAUT has been used in health care settings involving SRs [69], and its construct validity has been established through confirmatory factor analysis [70]. In this sample, the UTAUT subscales demonstrated excellent internal consistency for performance expectancy (Cronbach α=0.97), effort expectancy (Cronbach α=0.96), social influence (Cronbach α=0.87), and behavioral intention (Cronbach α=0.91), and questionable internal consistency for facilitating conditions (Cronbach α=0.60).

### Statistical Analysis

Analyses were conducted in IBM SPSS (version 29). As missing data were minimal (0%‐8% per variable) and assumed missing at random (Little’s Missing Completely At Random test: *χ*²_1387_=1415.3; *P*=.29), we used the semiparametric multiple imputation (m=5) procedure [71] to address missing values in the psychological questionnaires. Descriptive statistics were calculated for demographic, clinical, and psychological variables. Continuous variables were reported as means (SDs), and categorical variables were reported as frequencies and percentages. Pearson correlations examined bivariate variable relationships, following Hemphill’s guidelines for correlation interpretation: <0.20 (weak), 0.20 to 0.30 (moderate), and >0.30 (strong) [72]. Significant associations were entered in multivariable linear regression models to examine the association between psychological variables and UTAUT, controlling for sex, age, and smart technology experience. Depressive symptoms (PHQ-9) and generalized anxiety symptoms (GAD-7) were included to evaluate whether PPWB (BIT) predicted variance in SR acceptability independently of psychological distress. Urbanicity and CCI were included as covariates because they were relevant predictors of acceptability in prior analyses of this cohort [31]. Significance was set at *P*=.05. Unstandardized B coefficients with 95% CIs, *t* values, and effect sizes (*R²* and adjusted *R²*) were reported, along with variance inflation factor and tolerance statistics to assess multicollinearity. Model fit was evaluated using *F*-statistics and corresponding *P* values for each imputed dataset, as pooled omnibus test statistics are not provided in SPSS for multiply imputed data. Effect sizes were interpreted as small (*R²*=0.02), medium (*R²*=0.13), and large (*R²*=0.26) based on established guidelines [73,74].

In post hoc analyses, we conducted Pearson correlations between each BIT item and UTAUT behavioral intention to identify the item most strongly driving the association. This approach was used because the American Heart Association statement [43] recommends analyzing specific PPWB components, while the BIT provides only a summary score. Additionally, we examined exploratory Pearson correlations between individual BIT items and 25 rated SR capabilities and features to explore potential links between distinct aspects of psychological well-being and feature-specific acceptability, thereby generating preliminary insights relevant to SR design. Given the exploratory nature of these analyses and the large number of comparisons, they were not intended for formal significance testing. Finally, we repeated the multiple regression analyses using HF type (HFrEF and HF with preserved ejection fraction), LVEF, and NYHA class as alternative indicators of HF symptom burden, instead of the CCI, to explore their potential influence on the association between BIT and UTAUT behavioral intention.

## Results

### Sample Description

The study included 101 patients with HF (women: n=36, 36% female; and White: n=90, 89%), with a mean age of 68 (SD 10) years, and 33% (n=33) resided in urban communities (Table 1 and Multimedia Appendix 2). The average LVEF was 37.0 (SD 13.1), and the majority of patients were diagnosed with HFrEF (77/98, 76%) and classified as NYHA class II (77/101, 76%). The mean comorbidity index was 5.5 (SD 2.0), and the average time since diagnosis was 5.7 (SD 10.0) years.

**Table 1.** Participant characteristics of adults with heart failure (N=101).

Variables	Values
Age*,* mean (SD)	68.0 (9.9)
Female, n (%)	36 (35.6)
Race^g^, n (%)	
White	90 (89.1)
Arab	2 (2.0)
Black	2 (2.0)
Aboriginal	1 (1.0)
Chinese	1 (1.0)
Filipino	1 (1.0)
South Asian	1 (1.0)
Other	2 (2.0)
Marital status, n (%)	
Married or partner	70 (69.3)
Divorced or separated	10 (9.9)
Widowed	6 (5.9)
Single	15 (14.9)
Highest education, n (%)	
High school	23 (22.8)
Trade or college degree	37 (36.6)
Bachelor’s	28 (27.7)
Master’s or doctorate	13 (12.9)
Employment status, n (%)	
Employed	13 (12.9)
Unemployed, disability, or other	18 (17.9)
Retired	70 (69.3)
Community, n (%)	
Rural	28 (27.7)
Urban	33 (32.7)
Suburban	33 (32.7)
Mixed	7 (6.9)
Would likely use SR^b^, n (%)	67 (69.1)
Smart devices in use, mean (SD)	4.5 (2.2)
HFrEF^c^, n (%)	77 (76.2)
LVEF^d^, mean (SD)	37.0 (13.1)
NYHA^e^ class II, n (%)	77 (76.2)
Years since diagnosis, mean (SD)	5.7 (10.0)
CCI^f^, mean (SD)	5.5 (2.1)

a Race data were missing for 1 participant.

b SR: social robot.

c HFrEF: heart failure with reduced ejection fraction.

d LVEF: left ventricular ejection fraction.

e NYHA: New York Heart Association.

f CCI: Charlson Comorbidity Index.

### Experience With Technology

Most of the 101 participants reported using at least one smart device, including smartphones (n=87, 86%), laptops (n=69, 68%), iPads or tablets (n=54, 54%), or smart televisions (n=57, 56%). Fewer patients used wearable devices, such as smartwatches (n=31, 31%), voice assistants (eg, Google Home and Alexa; n=30, 30%), and smart medical or health-related sensors (eg, respiratory rate monitors, sleep monitors, heart rate sensors, and fall detection; n=21, 21%). The smart technology experience, indicated by the mean number of smart devices reported, was 4.5 (SD 2.2; range 0‐12).

### General Attitudes Toward SRs

As previously reported, more than half of the participants (53/99, 54%) reported being not at all or only somewhat aware of SRs before participating in this study, and 93% (92/99) had never interacted with an SR in the past [31]. In terms of usefulness, 58% (57/98) agreed or somewhat agreed that an SR would be helpful in their current health situation, and 69% (67/97) agreed or somewhat agreed that they would use an SR if one were available to them. Additional information regarding the samples’ preferred SR capabilities may be found elsewhere [31].

### Psychological, Personality, and Technology Acceptance Measures

The mean PHQ-9 score was 6.4 (SD 5.5), with 23% (23/101) of patients scoring in the clinical range (ie, above the cutoff for moderate depressive symptoms). The mean GAD-7 score was 4.8 (SD 5.1), with 17% (17/101) of patients crossing the threshold for clinically significant anxiety symptoms. Patients reported a moderate BIT score (mean 36.9, SD 7.1). Among the personality traits, conscientiousness (mean 11.1, SD 2.4) and agreeableness (mean 10.7, SD 2.3) received the highest ratings, followed by emotional stability (mean 10.1, SD 3.0), openness to experiences (mean 9.8, SD 2.5), and extraversion (mean 7.8, SD 2.8). Scores on the UTAUT subscales showed moderate acceptability of SRs in terms of performance expectancy (mean 17.0, SD 7.3; scale range 4‐28), social influence (mean 15.4, SD 5.6; scale range 4‐28), and facilitating conditions (mean 20.6, SD 4.2; scale range 4‐28). Acceptability was high regarding effort expectancy (mean 21.4, SD 5.6; scale range 4‐28). The behavioral intention to use SRs was moderate (mean 13.7, SD 4.9; scale range 3‐21; Table 2).

**Table 2.** Correlation matrix (Pearson *r* and two tailed *P*-value) of questionnaire scores assessing technology acceptability, mental health symptoms, psychological well-being, and personality traits in adults with heart failure (N=101).^a^

Variable	UTAUT^b^ performance	UTAUT effort	UTAUT conditions	UTAUT social	UTAUT behavioral intention	PHQ-9^c^	GAD-7^d^	BIT^e^	TIPI^f^ – Extraversion	TIPI – Agreeableness	TIPI – Conscientiousness	TIPI - Emotional Stability	TIPI - Openness to Experiences
UTAUT performance													
*r*	1	.505	.383	.684	.783	.164	.170	.083	-.072	-.071	-.050	-.104	-.035
*P* value	—	<.001	<.001	<.001	<.001	.11	.09	.41	.48	.49	.63	.30	.74
UTAUT effort													
*r*	.505	1	.745	.448	.597	-.090	-.060	.234	-.077	.124	-.013	.033	.069
*P* value	<.001	—	<.001	<.001	<.001	.38	.57	.02	.45	.25	.90	.74	.55
UTAUT conditions													
*r*	.383	.745	1	.337	.518	-.045	-.057	.263	.019	.033	-.024	-.043	.144
*P* value	<.001	<.001	—	.002	<.001	.66	.58	.008	.85	.76	.83	.69	.22
UTAUT social													
*r*	.684	.448	.337	1	.634	.013	.047	.238	-.029	.012	-.023	.080	-.049
*P* value	<.001	<.001	.002	—	<.001	.90	.65	.03	.78	.91	.82	.45	.65
UTAUT behavioral intention													
*r*	.783	.597	.518	.634	1	.031	.136	.198	-.026	-.015	-.001	-.057	.003
*P* value	<.001	<.001	<.001	<.001	—	.77	.21	.049	.80	.89	.99	.59	.98
PHQ-9													
*r*	.164	-.090	-.045	.013	.031	1	.756	-.554	-.257	-.238	-.388	-.551	-.136
*P* value	.11	.38	.66	.90	.77	—	<.001	<.001	.01	.02	<.001	<.001	.18
GAD-7													
*r*	.170	-.060	-.057	.047	.136	.756	1	-.399	-.237	-.196	-.237	-.629	-.193
*P* value	.09	.57	.58	.65	.21	<.001	—	<.001	.02	.05	.02	<.001	.06
BIT													
*r*	.083	.234	.263	.238	.198	-.554	-.399	1	.231	.264	.330	.349	.199
*P* value	.41	.02	.008	.03	.049	<.001	<.001	—	.02	.01	.001	<.001	.048
TIPI – Extraversion													
*r*	-.072	-.077	.019	-.029	-.026	-.257	-.237	.231	1	.032	.153	.212	.182
*P* value	.48	.45	.85	.78	.80	.01	.02	.02	—	.76	.13	.04	.07
TIPI – Agreeableness													
*r*	-.071	.124	.033	.012	-.015	-.238	-.196	.264	.032	1	.230	.490	.259
*P* value	.49	.25	.76	.92	.89	.02	.05	.01	.76	—	.02	<.001	.01
TIPI – Conscientiousness													
*r*	-.050	-.013	-.024	-.023	-.001	-.388	-.237	.330	.153	.230	1	.314	.248
*P* value	.63	.90	.83	.82	.99	<.001	.02	.001	.13	.02	—	.002	.02
TIPI - Emotional Stability													
*r*	-.104	.033	-.043	.080	-.057	-.551	-.629	.349	.212	.490	.314	1	.242
*P* value	.30	.74	.69	.45	.59	<.001	<.001	<.001	.04	<.001	.002	—	.02
TIPI - Openness to Experiences													
*r*	-.035	.069	.144	-.049	.003	-.136	-.193	.199	.182	.259	.248	.242	1
*P* value	.74	.55	.22	.65	.98	.18	.06	.048	.07	.01	.02	.02	—

a Data were multiply imputed and pooled. Higher scores indicate greater levels of the respective construct.

b UTAUT: Unified Theory of Acceptance and Use of Technology.

c PHQ-9: Patient Health Questionnaire-9.

d GAD-7: Generalized Anxiety Disorder-7.

e BIT: Brief Inventory of Thriving.

f TIPI: Ten-Item Personality Inventory.

### Association of Psychological Variables With SR Acceptability

Table 2 displays the means and correlations between the study variables. PPWB (BIT score) was positively correlated with UTAUT effort expectancy (*r(99*)=0.234; *P*=.02), UTAUT facilitating conditions (*r(99*)=0.263; *P*=.008), UTAUT social influence (*r(99*)=0.238; *P*=.03), and UTAUT behavioral intention (*r(99*)=0.198; *P*=.049). There were no significant correlations between PHQ-9, GAD-7, and TIPI with any of the UTAUT subscales.

Across the original and imputed datasets, the overall regression model predicting performance expectancy was not statistically significant (*F*_8, 77-90_=1.24-1.55; all *P*>.15). The model predicting social influence was statistically significant in the original dataset (*F*_8,75_=2.21; *P*=.04) but not in any of the imputed datasets (*F*_8,90_=1.12-1.91; all *P*≥.07). In contrast, the models predicting effort expectancy, facilitating conditions, and behavioral intention were statistically significant across the original and all imputed datasets (effort expectancy: *F*_8,77-90_=3.11‐4.29; all *P*≤.004; facilitating conditions: *F*_8,76-90_=3.13‐3.66; all *P*≤.004; and behavioral intention: *F*_8,79-90_=2.20‐3.12; all *P*≤.04).

**Figure 1.**
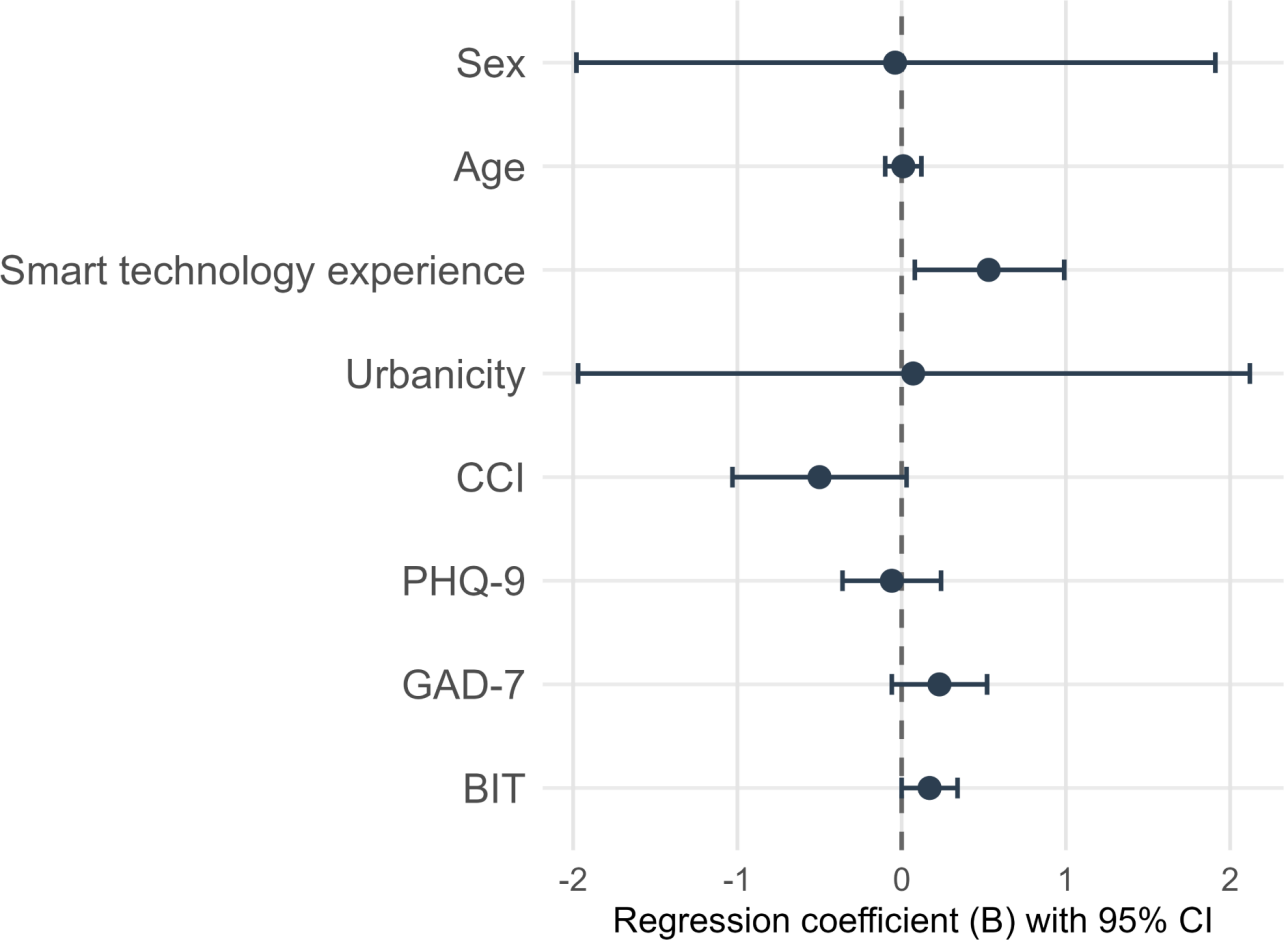
Forest plot of regression coefficients (B) and 95% CI for predictors of Unified Theory of Acceptance and Use of Technology behavioral intention. Sex: 0=male, 1=female; Smart technology experience=number of devices in use; Urbanicity: 0=nonurban, 1=urban; BIT: Brief Inventory of Thriving; CCI: Charlson Comorbidity Index; GAD-7: Generalized Anxiety Disorder-7; PHQ-9: Patient Health Questionnaire-9.

In the multiple regression models (Tables3  4, Figure 1, Supplement S3-S6 in Multimedia Appendix 1), higher BIT scores were associated with increased SR acceptability, including UTAUT facilitating conditions (*B*=0.17; *P*=.01) and behavioral intention (*B*=0.17; *P*=.04). All models were adjusted for sex, age, smart technology experience, urbanicity, CCI, depressive, and anxiety symptoms.

**Table 3.** Multiple regression analyses examining the association of psychological well-being (Brief Inventory of Thriving [BIT]) with technology acceptability (Unified Theory of Acceptance and Use of Technology [UTAUT]) adjusted for covariates in adults with heart failure (N=101).^a^

	UTAUT performance expectancy						UTAUT effort expectancy							UTAUT facilitating conditions						
Predictors	B (SE)	95% CI	*t* ^b^	*P* value	Tolerance	VIF^c^	B (SE)		95% CI	*t*	*P* value	Tolerance	VIF	B (SE)		95% CI	*t*	*P* value	Tolerance	VIF
Sex^d^	-0.64 (1.60)	-3.78, 2.50	-0.4	.9	0.91	1.1	-0.59 (1.09)		-2.72, 1.54	-0.54	.59	0.93	1.07	-0.63 (0.82)		-2.25, 0.98	-0.77	.44	0.92	1.08
Age	0.00 (0.09)	-0.17, 0.17	-0.03	.98	0.72	1.4	-0.14 (0.06)		-0.26, -0.02	-2.34	.02	0.71	1.41	-0.05 (0.05)		-0.14, 0.04	-1	.32	0.73	1.37
Smart technology experience^e^	0.36 (0.35)	-0.32, 1.04	1.03	.30	0.86	1.16	0.45 (0.24)		-0.03, 0.93	1.85	.06	0.87	1.15	0.48 (0.18)		0.12, 0.84	2.63	.009	0.87	1.16
Urbanicity^f^	1.13 (1.63)	-2.07, 4.32	0.69	.49	0.94	1.07	-1.23 (1.11)		-3.40, 0.94	-1.11	.26	0.93	1.07	-0.18 (0.88)		-1.91, 1.55	-0.2	.84	0.93	1.08
CCI^g^	-0.66 (0.43)	-1.49, 0.18	-1.55	.12	0.69	1.44	-0.24 (0.29)		-0.81, 0.33	-0.82	.41	0.69	1.46	-0.41 (0.22)		-0.85, 0.02	-1.88	.06	0.71	1.41
PHQ-9^h^	0.27 (0.24)	-0.20, 0.73	1.12	.26	0.34	2.91	-0.11 (0.16)		-0.43, 0.20	-0.7	.49	0.35	2.86	0.08 (0.12)		-0.16, 0.32	0.65	.52	0.34	2.94
GAD-7^i^	0.12 (0.22)	-0.31, 0.55	0.54	.59	0.42	2.36	0.02 (0.16)		-0.29, 0.32	0.11	.92	0.44	2.28	-0.05 (0.12)		-0.29, 0.18	-0.43	.66	0.42	2.37
BIT	0.23 (0.13)	-0.02, 0.48	1.84	0.07	0.63	1.58	0.16 (0.09)		-0.02, 0.33	1.75	.08	0.64	1.57	0.17 (0.07)		0.04, 0.30	2.49	.01	0.64	1.56

a Results are pooled estimates from multiple imputation; therefore, unstandardized regression coefficients ( B) are reported instead of standardized betas (β). Each B represents the expected change in the outcome score for a 1-unit increase in the predictor. Model fit ( F-statistics and P values) varied across imputed datasets and is therefore summarized in the text rather than pooled in the table, as SPSS does not provide pooled omnibus test statistics.

b Degrees of freedom for t values are not reported in the table, as they are estimated under Rubin’s rules for multiple imputation and vary across imputations.

c VIF: variance inflation factor.

d Sex: 0=male, 1=female.

e Smart technology experience=number of devices in use.

f Urbanicity: 0=nonurban and 1=urban.

g CCI: Charlson Comorbidity Index.

h PHQ-9: Patient Health Questionnaire-9.

i GAD-7: Generalized Anxiety Disorder-7.

**Table 4.** Multiple regression analyses examining the association of psychological well-being (Brief Inventory of Thriving [BIT]) with technology acceptability (Unified Theory of Acceptance and Use of Technology [UTAUT]) adjusted for covariates in adults with heart failure (N=101).^a^

	UTAUT social influence						UTAUT behavioral intention					
Predictors	B (SE)	95% CI	*t* ^b^	*P* value	Tolerance	VIF^c^	B (SE)	95% CI	*t*	*P* value	Tolerance	VIF
Sex^d^	-0.33 (1.19)	-2.67, 2.01	-0.28	.78	0.92	1.08	-0.04 (0.99)	-1.98, 1.91	-0.04	.97	0.92	1.09
Age	0.10 (0.07)	-0.03, 0.23	1.49	.14	0.72	1.38	0.01 (0.06)	-0.10, 0.12	0.18	.86	0.73	1.38
Smart technology experience^e^	-0.04 (0.27)	-0.57, 0.48	-0.17	.87	0.86	1.17	0.53 (0.23)	0.08, 0.99	2.3	.02	0.87	1.15
Urbanicity^f^	-0.07 (1.20)	-2.42, 2.28	-0.06	.95	0.93	1.08	0.07 (1.04)	-1.97, 2.12	0.07	.94	0.93	1.08
CCI^g^	-0.29 (0.32)	-0.91, 0.34	-0.89	.37	0.7	1.42	-0.50 (0.27)	-1.03, 0.03	-1.84	.07	0.7	1.42
PHQ-9^h^	0.17 (0.18)	-0.18, 0.52	0.95	.34	0.33	3	-0.06 (0.15)	-0.36, 0.24	-0.37	.71	0.34	2.92
GAD-7^i^	0.06 (0.17)	-0.26, 0.39	0.38	.70	0.41	2.47	0.23 (0.15)	-0.06, 0.52	1.57	.12	0.43	2.35
BIT	0.28 (0.10)	0.07, 0.48	2.8	.008	0.65	1.54	0.17 (0.09)	0.00, 0.34	2.02	.04	0.64	1.57

a Results are pooled estimates from multiple imputation; therefore, unstandardized regression coefficients ( B) are reported instead of standardized betas (β). Each B represents the expected change in the outcome score for a 1-unit increase in the predictor. Model fit ( F-statistics and P values) varied across imputed datasets and is therefore summarized in the text rather than pooled in the table, as SPSS does not provide pooled omnibus test statistics.

b Degrees of freedom for *t* values are not reported in the table, as they are estimated under Rubin’s rules for multiple imputation and vary across imputations.

c VIF: variance inflation factor.

d Sex: 0=male, 1=female.

e Smart technology experience=number of devices in use.

f Urbanicity: 0=nonurban and 1=urban.

g CCI: Charlson Comorbidity Index.

h PHQ-9: Patient Health Questionnaire-9.

i GAD-7: Generalized Anxiety Disorder-7.

In addition, 2 covariates showed significant associations with UTAUT subscales. Age was negatively associated with effort expectancy (*B*=−0.14; *P*=.02). Smart technology experience was positively associated with facilitating conditions (*B*=0.48; *P*=.009) and behavioral intention (*B*=0.53; *P*=.02).

### Post Hoc Analyses

Among the 10 items of the BIT, UTAUT behavioral intention to use an SR was positively correlated with *“*I am achieving most of my goals” (*r*(99)=0.233; *P*=.02) and “I am optimistic about my future*”* (*r*(99)=0.210; *P*=.04), while correlations with “My life has a clear sense of purpose*”* (*r*(99)=.189; *P*=.07), “What I do in life is valuable and worthwhile*”* (*r*(99)=0.184; *P*=.07), “I can succeed if I put my mind to it*”* (*r*(99)=0.142; *P*=.17), “I feel good most of the time*”* (*r*(99)=0.139; *P*=.17), “My life is going well” (*r*(99)=0.108; *P*=.29), “In most activities I do, I feel energized” (*r*(99)=0.092; *P*=.37), “I feel a sense of belonging in my community” (*r*(99)=0.124; *P*=.24), and “There are people who appreciate me as a person*”* (*r*(99)=0.073; *P*=.48) were small and not statistically significant.

The BIT total score was associated with the SR feature “Entertainment” (*r*(93)=0.216). Among individual items, “I feel a sense of belonging to my community” was linked to the largest number of SR features (Supplement S7 in Multimedia Appendix 1). Including HF type, LVEF, or NYHA class as control variables for symptom burden (instead of CCI) in the multiple regression analyses did not affect the association between BIT and UTAUT behavioral intention.

## Discussion

### Principal Findings

This study is a pioneering study to investigate psychological factors associated with determinants of SR acceptability in an HF patient sample. The findings highlight that PPWB is associated with dimensions of patients’ SR acceptability, independent of psychological distress: patients with higher PPWB are more likely to feel assured in their personal resources and knowledge to operate SRs and more likely to intend to use an SR in the future. These findings extend our earlier research, which demonstrated that urbanicity and lower comorbidity levels are linked to higher SR acceptability [31], positioning PPWB as a key psychological factor, alongside demographic and clinical variables, in influencing patients’ willingness to adopt SRs. This is consistent with broader HF literature linking PPWB to greater adherence to health behaviors [41,42] and increased self-care [75], underscoring the relevance of psychological well-being in HF management. Taken together, these findings suggest that patients with higher PPWB may not only be more motivated to manage their condition actively but also more receptive to emerging tools that support autonomy and self-efficacy in care.

In particular, the BIT items, “I am achieving most of my goals” and “I am optimistic about my future” exhibited the strongest associations with patients’ intention to use an SR. This indicates that a forward-looking, goal-oriented mindset may increase openness to innovative health care tools such as SRs. In line with this, a previous study found that satisfaction with life was associated with higher SR acceptability in healthy adults [34]. Moreover, research in general populations has indicated that positive emotions, such as enjoyment and excitement, are linked to a higher willingness to engage with SRs [36,76] and a higher confidence regarding one’s ability to use SRs [38]. In our HF population, an optimistic outlook on life seems to be a more significant predictor of SR acceptability than momentary emotional states. This may be explained by our participants not directly interacting with SRs in a trial setting but instead simply expressing their attitudes toward SRs. Further research is needed to explore how different PPWB components influence SR acceptability in HF populations.

Interestingly, although 23% (23/101) of participants had at least moderate depressive symptoms and 17% (17/101) at least clinically elevated anxiety symptoms, neither depression nor anxiety scores were significantly associated with determinants of SR acceptability. This finding is consistent with a study in healthy older adults, where depressive symptoms were not associated with SR acceptability [34]. Similarly, in students, negative emotions such as fear and anxiety were either weakly or not at all related to SR acceptability or willingness to interact with SRs in laboratory settings [36]. While some research has linked anxiety to lower perceived ease of use of SRs [38], we found no such relationship. Conversely, studies examining loneliness, which is often associated with depressive symptoms [77], have found it to be positively correlated with SR acceptability, particularly during the COVID-19 pandemic [78]. Notably, in our prior study, few patients with HF expressed interest in companionship features; instead, most were drawn to SRs for functional tasks such as monitoring (eg, blood pressure tracking) and managing appointments [31]. This suggests that SR features focusing on reminders, monitoring, and educational support may be more appealing to patients with HF than those designed for social interaction or mental health.

Unexpectedly, personality traits, such as agreeableness, extraversion, and openness, did not show a significant association with SR acceptability in our HF sample. In contrast, Esterwood et al [51] reported in their meta-analysis that individuals who are more agreeable, extroverted, and open are more likely to accept SRs, but they also acknowledged that this link weakens in older populations. Our sample included older adults, and if age indeed moderates these associations (with weaker associations between personality and SR acceptability with age) as suggested by the findings of Esterwood et al [51] and Kim et al [79], this may help understand our null effect. Alternatively, the low internal consistency of the 2-item TIPI subscales in our sample (α=.33-.40) may have contributed to measurement error, and the null associations with personality traits should be interpreted with caution.

Notably, the general acceptability of SRs in this sample was high, with 69% (67/97) of participants indicating they would likely use an SR if available. This level is similar to a small sample of patients in cardiac rehabilitation [29]. This suggests that SR technology is acceptable in patients with cardiac conditions. Additionally, our investigation into HF health care providers’ perspectives similarly revealed openness to SRs in roles such as data collection, education, and patient prompts but highlighted hesitancy regarding interventional applications [30].

In light of these findings, when considering the integration of SRs into HF care, clinicians should be aware that patients’ PPWB, particularly their sense of optimism and goal-directedness, may significantly influence acceptance. Assessing PPWB may help identify patients most receptive to such technologies and guide tailored implementation strategies. Co-designing SR features in collaboration with patients, health care providers, and developers, guided by intervention mapping [80,81], can ensure that SRs are functionally relevant and emotionally engaging. Our previous findings suggest that patients with HF living in urban areas with low comorbidities may be ideal candidates for testing an initial SR prototype [31]. This study further indicates that patients with high PPWB may be more likely to engage with and benefit from SRs. In particular, SR features such as self-tracking, proactive health alerts, and especially gamification could empower these individuals to maintain their well-being [82]. Consistent with this, the PPWB score was associated with the SR feature “Entertainment,*”* highlighting the potential motivational role of enjoyable, engaging interactions. The fact that *“*I feel a sense of belonging in my community*”* was linked to the largest number of SR features indicates that social connectedness may be a key driver of engagement across multiple SR functions.

Our findings also suggest that individual characteristics related to age and technology familiarity may influence patient acceptability of SRs. Specifically, higher perceived effort among older participants highlights potential age-related barriers to adoption. In contrast, greater smart technology experience appears to facilitate both perceived resources and intentions to use. These findings suggest that SRs should be designed to minimize complexity and cognitive load to better support older users, while also incorporating intuitive interfaces, clear guidance, and accessible support features to accommodate individuals with limited prior experience with digital technologies. For patients experiencing emotional distress or lower levels of positive affect, SRs may benefit from simplified, nonintrusive designs that provide instrumental support without adding cognitive burden [9]. For patients who are skeptical of SRs, controlled environments where they can test the technology could help alleviate concerns and build trust [83]. Research has shown that hands-on interaction with SRs can significantly increase acceptance of the technology [84]. On the basis of our findings, relevant features for patients appear to include monitoring and appointment scheduling, which could be prioritized for initial testing [31].

Future research should explore the longitudinal effects of SR use on emotional well-being and health outcomes, providing crucial insights into the sustainability and broader impact of SR integration in HF care. Longitudinal studies will also be essential in tracking how SR acceptance evolves over time and throughout the course of illness, identifying the key determinants of sustained use, which is a central challenge in SR applications [85]. By advancing collaborative and patient-centered design processes, SR technologies can be optimized not only to meet the functional demands of HF management but also to foster meaningful patient engagement.

### Limitations

First, the relatively small sample size may limit the generalizability of our findings to the broader HF population. In our sample, only 36% (36/100) were women, which is consistent with previous HF studies [86], but slightly lower than the expected 41% (41/100) of women in the overall HF population. While prior research suggests that men are generally more likely than women to engage with technology and report higher technology acceptance in older adult populations [87], these differences appear to be minimal or may even be reversed in the context of SRs [88,89]. As such, the underrepresentation of women may influence the generalizability of the acceptability estimates observed in this study, although the direction and magnitude of any such effect remain uncertain. The sample was also predominantly White (90/101, 90%), which may limit generalizability across ethnic and cultural groups [90]. Structural, social, and cultural factors, such as access to technology, familiarity with digital tools, and culturally shaped attitudes toward robots, could influence both the perceived ease of use and the support available for technology adoption, potentially altering the relationships observed in this study. These considerations underscore the need for more inclusive recruitment strategies to ensure that the perspectives and needs of diverse populations are adequately represented in future research [91].

Second, the predominance of NYHA class II patients in our sample raises the possibility that individuals with more advanced HF and greater symptom burden may experience lower levels of PPWB, such as reduced optimism or sense of control, which could in turn affect their openness to adopting SR technology. Patients with more severe HF may also face greater challenges in using technology, perceive higher effort to engage with SRs, and require additional support for adoption. However, *post hoc* analyses revealed that HF type, LVEF, and NYHA class did not significantly influence the relationship between PPWB and SR acceptability, suggesting that the observed associations may be consistent across different clinical presentations.

Third, the time since diagnosis showed a right-skewed distribution, which should be considered when interpreting summary statistics. Furthermore, the TIPI subscales showed low internal consistency (α=.33-.40), which may have contributed to measurement error and warrants cautious interpretation of null findings. While the TIPI was chosen for brevity and minimal participant burden, future studies could use more comprehensive personality assessments.

Fourth, the videos patients watched before completing the questionnaires may have influenced their responses, potentially creating a framing effect, where marketing-style presentation could inflate perceptions of performance expectancy and minimize perceived effort expectancy compared to real-world use. However, this introduction was essential, as 93% (92/99) of patients were unfamiliar with SRs. As this was early-phase research with limited funding, we opted not to provide a demonstration of a physical robot. It is possible that patients’ perspectives on using SRs might have been different if they had the opportunity to interact directly with an SR.

Finally, as the study used a cross-sectional design, temporal relationships cannot be discerned. While emotional states such as PPWB may be key predictors of SR acceptability, it remains unclear how these factors influence long-term usage. Longitudinal studies could assess whether patients with high PPWB not only accept SRs more readily but also maintain consistent engagement over time. Importantly, this study is the first to investigate the association between psychological factors and SR acceptability among patients with HF, setting the stage for future longitudinal research and the co-design of SR interventions tailored to the unique needs of HF care.

### Conclusions

This study highlights the role of PPWB in relation to determinants of SR acceptability among patients with HF, while negative psychological factors and personality traits showed no significant associations. These findings emphasize the importance of considering PPWB when designing and implementing SR interventions in HF care. Patients with high PPWB may be ideal candidates for testing an initial SR prototype, as they are more likely to engage with and benefit from SRs.

## Supplementary material

10.2196/83163 Multimedia Appendix 1 Video descriptions and transcripts, study questionnaires, and additional tables and figures supporting the main analyses.

10.2196/83163 Multimedia Appendix 2 Graphical abstract.

## Abbreviations

BIT: Brief Inventory of Thriving
CCI: Charlson Comorbidity Index
GAD-7: Generalized Anxiety Disorder-7 scale
HF: heart failure
HFrEF: Heart failure with reduced ejection fraction
LVEF: left ventricular ejection fraction
NYHA: New York Heart Association
PHQ-9: Patient Health Questionnaire-9
PPWB: positive psychological well-being
REDCap: Research Electronic Data Capture
SR: social robot
TIPI: Ten-Item Personality Inventory
UTAUT: Unified Theory of Acceptance and Use of Technology
