# Supplementary material for: Positive Psychological Well-Being and Determinants of Social Robot Acceptability Among Patients With Heart Failure: Cross-Sectional Questionnaire Study
Source: JMIR Cardio. 2026 Jun 2;10:e83163. doi: 10.2196/83163 (PMC13229465; doi:10.2196/83163)
Supplement: Multimedia Appendix 1 [file cardio-v10-e83163-s001.pdf]

Supplemental Material

Title: Thriving with technology: Positive psychological well-being is associated with determinants of social robot acceptability among patients with heart failure

Lisa-Marie Maukel (PhD), Karen Bouchard (PhD), Jess G. Fiedorowicz (MD, PhD), Kerstin Dautenhahn (Dr. rer. nat.), Moojan Ghafurian (PhD), Thais Coutinho (MD, FRCPC), Caroline McGuinty (MD, FRCPC), Peter P. Liu (MD, FRCPC), & Heather Tulloch (PhD, C. Psych)

Table of Contents

Supplement 1. Social Robot Video Descriptions and Transcripts
Supplement 2. Questionnaire General Attitudes toward Technology and Social Robots, and Desired Social Robot Capabilities
Supplement 3. Figure S3. Forest plot of regression coefficients (B) and 95% CI for predictors of UTAUT performance expectancy
Supplement 4. Figure S4. Forest plot of regression coefficients (B) and 95% CI for predictors of UTAUT effort expectancy
Supplement 5. Figure S5. Forest plot of regression coefficients (B) and 95% CI for predictors of UTAUT facilitating conditions
Supplement 6. Figure S6. Forest plot of regression coefficients (B) and 95% CI for predictors of UTAUT social influences
Supplement 7. Correlation Matrix of individual BIT items and 25 rated social robot capabilities and features

Supplement 1. Social Robot Video Descriptions and Transcripts

Video 1: Mabu — Personal Healthcare Companion (Catalia Health Inc.)

https://www.youtube.com/watch?v=mj-3bot3D34

Video length: 1:30 minutes

The video presents Mabu, a small, yellow tabletop personal healthcare robot designed to support patients managing chronic illness at home. The robot stands approximately 30 cm tall and features a friendly, expressive face with animated eyes that blink and make eye contact, creating a more human-like interaction. Below its head, Mabu has a tablet interface that displays options and dialogue.
In the video, the robot is positioned on a bedside desk, and a patient (seen from behind) interacts with the tablet. Selecting an option triggers brief, context-specific dialogue displayed on the screen. After this scene, the video shows images of two different patients (a young man and an older woman), each with corresponding interaction options popping up below their images, demonstrating how the robot personalizes interactions for different users. The video concludes with Mabu blinking at the camera.

Transcript

0:00 [Music]

0:05 Mabu: "Hi, I'm Mabu, a personal healthcare companion. My job is to keep patients engaged with their care over the long term and to gather data on a daily basis that's useful for their care. I speak out loud and show what I'm saying on my screen to make sure it's easy to converse with me. Patients can respond by speaking or by touching the buttons on my screen. I follow the patient's face so that I can make appropriate eye contact. 'How is it going with your medications?'"

0:39 Female voice: "It's okay."
Mabu: "Did you take them today?"

0:43 Female voice: "Only in the morning."
Mabu: "Are you facing any challenges?"

0:45 Female voice: "I just got busy and forgot."
Mabu: "Would you like me to remind you?"

0:48 Female voice: "Text me."
Mabu: "Okay, I will remind you tomorrow when it's time."
Female voice: "Great."

0:55 Mabu: "I learn about each patient over time. I tailor our conversations to their personality, learning what kind of support is both the most engaging and the most

effective. Because each patient is unique, so are their medical and treatment issues, and so are our conversations."

1:14 Female voice: "She says something new every day."

1:17 Mabu: "A typical conversation lasts only 2 or 3 minutes, but I can also check in at other times if I need to."

1:25 Female voice: "Thanks, Mabu."
[Music]

Video 2: PARO — Therapeutic Robotic Seal

https://www.youtube.com/watch?v=PAJ2GXzaJtQ&t=66s

Video length: 3:53 minutes

The video presents PARO, a baby harp seal–shaped therapeutic robot designed to provide emotional and social support, particularly for patients with chronic illness or cognitive impairments. PARO measures approximately 50 cm in length and features a soft, lifelike exterior with animated eyes and flippers.

The video is filmed in a residential home setting, where three dementia care specialists describe PARO's features. Residents are shown interacting with the robot, observing and petting it, and smiling while engaging with its movements and sounds. The video alternates between shots of the specialists speaking and the residents interacting with PARO.

Transcript

0:00 [Music]

0:06 HCP 1: “PARO is an electronic therapeutic seal, sounds a bit unusual. He was developed in Japan by Professor Shibata, who has actually come over and visited us and given us a full presentation about all of the things that PARO can do.”

0:19 HCP 2: “We can use PARO with residents that have responsive behaviors, including wandering or yelling. That can actually result in a calming effect on the resident. It helps reduce anxiety levels, depression, and behavioral problems.”

0:41 HCP 1: “We started at the Boulevard in [x]. We've used him now across all of our residential homes and home care services and we've had really positive responses. Both staff and residents really became quite attached to Sammy the seal.”

0:57 HCP 3: “You can't help but pet him. You just sit there, you're talking to him and petting him, and then you look at yourself and say, ‘Oh look at me, I'm talking to a robot.’”

1:11 Resident 1: “Haven't you had your lunch? Haven't you had your lunch? Even nodded your head, even nodded your head. No, no, it's just lovely. Can I nurse you for a little bit?”

1:24 HCP 2: “You certainly can.”

1:27 Resident 1: “Alright, you have a sleep. And I'll think of something.”

1:29 HCP 3: “It's such an engaging strategy for us. We can engage someone who might be getting agitated, might be wandering, might be feeling sad. He's such a fun thing—he smiles at you, and with his artificial intelligence, you start to think that he's really listening to you.”

1:47 Resident 1: “I am being happy with you, yes, Sammy. You almost feel he’s answering.”

1:54 HCP 2: “What do you find most enjoyable spending time with Sammy?”
Resident 1: “Well, everything. What else can I say when I'm nursing you? What else can I say?”

2:12 HCP 2: “I find with a lot of residents, they're amazed with these sounds and his movements. They're intrigued with his beautiful big black eyes; they enjoy his softness.”

2:24 Resident 1: “And the eyes are just so beautiful. Look, for goodness’ sake, let me sleep, yeah.”

2:34 HCP 2: “And some nurture him like a young child. I've even had one resident sing a beautiful song to him.”

2:40 HCP 1: “In particular, there was one lady who was actually quite agitated, walking around a lot trying to leave. When we took Sammy to her, she took him straight from us, sat down with him, and started to pet and really talk to him. Not only the socialization between herself and PARO, but then other residents became involved as well and started talking as a group. It's one of the best responses I've seen, and I've worked in dementia care for many years.”

3:07 HCP 3: “You just can't help but love him. You can't help but actually want to talk to him, want to pet him. You think he's going to give you an answer—if only he could talk, he would actually give you all the answers that you needed—but he can be a best friend; you can tell him everything.”

3:22 HCP 1: “Our experience of using new technologies is that it really promotes well-being: people's mental health, their physical well-being, and their social well-being. The technology provides opportunities for people to experience these with care staff, with lifestyle staff, and we just want to continue to offer as many possibilities as possible for people to get these experiences.”

[Music]

Video 3: Elli-Q — Social Companion Robot (Intuition Robotics / Fuseproject)

https://www.youtube.com/watch?v=6otrRef6i8A

Video length: 1:56 minutes

Elli-Q is a sleek, tablet-integrated social companion robot with a movable display mount that allows subtle head movements to convey attention and responsiveness. Its minimalist design focuses on gestural and interactive cues rather than human-like facial features. The tablet provides visual prompts and interactive content, while Elli-Q's speech and gestures create a lifelike, engaging, and socially supportive presence.

The video begins with text slides:

- "50% of women aged 75+ live alone"
- "People aged 60+ commonly report feelings of loneliness"
- "Technology can complicate the way we communicate. ElliQ simplifies how older adults connect with the world"
- "Technology can complicate the way we communicate. ElliQ simplifies how older adults connect with friends"
- "Technology can complicate the way we communicate. ElliQ simplifies how older adults connect with family"

Next, an older woman (Mary) is seated on a couch reading a magazine, with Elli-Q positioned on a desk beside her. Elli-Q begins speaking:

0:23 Elli-Q: "Mary, Megan sent a new photo. Would you like to look at it?"

0:28 Mary: "Yes, please. Oh, he's a gem!"

0:31 Elli-Q: "Would you like to respond to Megan's post?"

0:36 Mary: "Sure"

Following this, another text slide appears:

- "ElliQ helps you get things done and keeps you engaged"
-

Mary then responds verbally to Elli-Q's prompts:

0:39 Mary: "ElliQ reminds me to take my meds, arranges rides for me, she even reminds me of all my appointments."

0:44 Elli-Q: "Mary, don't forget Bridge with the Golden Girls at 1:00 p.m. Would you like to practice?"

0:50 Mary: "Oh, I don't need to practice."

0:54 Elli-Q: “I didn't catch that, do you want to play bridge or not?”

0:54 Mary: “Oh fine, let's play”

Another slide appears:

- “ElliQ allows you to share information with loved ones… but only if you want to”
-

Mary is then shown doing Tai Chi while interacting with Elli-Q:

1:03 Elli-Q: “Mary, you wanted to Skype with Liz. Would you like to do that now?”

1:08 Mary: “Oh sure, that sounds great… hey honey”

1:15 Liz (on Elli-Q tablet): “Hi Mom. Hey, I just noticed—is it a little cool in the house?”

1:20 Mary: “Oh it feels great. I'm doing my Tai Chi now.”

1:23 Liz: “Yeah, I can see. Okay Mom, just checking in. You take care.”

1:27 Mary: “I'm fine, sweetie. Chat later.”

Final text slide appears:

- “ElliQ proactively suggests personalized activities and digital content”
-

Elli-Q suggests activities:

1:34 Elli-Q: “There's a new TED Talk waiting for you if you'd like to watch it, or perhaps you could go for a walk.”

1:41 Mary: “That's a good idea, Elli-Q.”

1:44 Elli-Q: “Great Mary, I'll be right here when you get back.”

1:55 Mary: “Great”

Video 4: Pepper — Humanoid Social Robot (SoftBank Robotics)

https://www.youtube.com/watch?v=XlDPMA6mn1g

Video length: 0:52 minutes

Pepper is a 1.20 m tall humanoid social robot with a touchscreen display on its chest, articulated head and arms, and a wheeled base for autonomous movement. Designed for natural human interaction, Pepper integrates speech recognition, facial recognition, and emotion analysis to engage in conversation, respond to users' expressions and vocal tones, and convey social presence through gestures and motion. Its design emphasizes approachability and a non-threatening human-like form, supporting communication and engagement in social settings.

In the video, an older woman (Rebecca) encounters Pepper in front of a pink screen background.

Transcript

0:00 Rebecca: "Oh my lord, what—what's that?"

0:09 Pepper: "Hello, my name is Pepper. I'm here at your service."

0:12 Rebecca: "Are you well? Do you know Dr. Mitchell, my doctor?"

0:20 Pepper: "Dr. Robert Mitchell? Would you like me to book an appointment for you?"

0:22 Rebecca: "That would be brilliant. I am Rebecca Parker."

0:26 Pepper: "He is available at 11:30."

0:29 Rebecca: "That would be perfect; it's just before lunch."

0:31 Pepper: "Yay! Appointment confirmed. Don't forget to do your daily exercises."

Slide: "Here to help and to care"

0:43 Rebecca: "Pepper, it seems I have a new friend."

0:44 [Music]

Supplement 2. Questionnaire General Attitudes toward Technology and Social Robots, and Desired Social Robot Capabilities

General Views on Technology

How would you rate your interest towards interacting with new technologies?

- ☐ Very interested
- ☐ Moderately interested
- ☐ Somewhat disinterested
- ☐ Not interested

How do you normally react to new technology-related services or products?

- ☐ Always adopt
- ☐ Adopt most of the time
- ☐ Adopt occasionally
- ☐ Almost never adopt

Do you feel that you have support from others when learning a new technology?

- ☐ Completely supported
- ☐ Moderately supported
- ☐ Somewhat supported
- ☐ Not at all supported

Please select what type of smart devices do you currently have?

- ☐ Smart phone
- ☐ iPad
- ☐ Laptop
- ☐ Desktop computer
- ☐ Smart Television
- ☐ Voice assistant agent or a robot (e.g., Google Home, Alexa, Jibo, Vector, etc.)
- ☐ Vacuuming or Mopping Robots, or a robotic lawn mower (e.g., Rumba, Braava Jet, Shark IQ, etc.)
- ☐ Home Security systems, or cameras for monitoring people/ pets (e.g., security cameras, contact sensors, motion detectors, cameras for monitoring a child, pet, etc.)
- ☐ Smart Smoke, carbon monoxide (co), or water leak detectors, etc. (e.g., Google Nest Alarm, etc.)
- ☐ Smart home appliances (e.g., smart fridge, such as a fridge with a tablet that has voice assistants)
- ☐ Smart medical or health related sensors (e.g., respiratory rate monitor, sleep monitor, heart rate sensors, fall detection, etc.)
- ☐ Wearable devices (e.g., fit bit, smart watch, etc.)
- ☐ Other: ______________________

Study ID: ____________________
Date: ______________________

General Views of Social Robots

How would you rate your awareness of social robots prior to participating in this study?
- ☐ Very aware
- ☐ Aware
- ☐ Not very aware
- ☐ Not aware at all

Have you interacted with a social robot in the past?
- ☐ Yes
- ☐ No

How important is it that a social robot has realistic human traits?
- ☐ Very important
- ☐ Important
- ☐ Not very important
- ☐ Not important at all

How important is it that a social robot is capable of expressing emotions?
- ☐ Very important
- ☐ Important
- ☐ Not very important
- ☐ Not important at all

A social robot would be useful to me in my current situation
- ☐ Agree
- ☐ Somewhat agree
- ☐ Somewhat disagree
- ☐ Disagree

I would use a social robot if it were to become available to me
- ☐ Agree
- ☐ Somewhat agree
- ☐ Somewhat disagree
- ☐ Disagree

In a few statements, how would you describe your general feelings towards social robots?

Study ID: ____________________
Date: ______________________

Social Robot Capabilities

Please rank the usefulness of the following social robot capabilities to provide care for you (or your loved one) at home. Please circle the number appropriate to your answer, using the following rating scale:

Extremely useless	Somewhat useless	Neither useless nor useful (neutral)	Somewhat useful	Extremely useful
1	2	3	4	5

1. Scheduling appointments	1	2	3	4	5
2. Facilitating a telemedicine interview	1	2	3	4	5
3. Reminders to take medication, exercise, or eat	1	2	3	4	5
4. Monitoring changes in mood	1	2	3	4	5
5. Detecting falls	1	2	3	4	5
6. Monitoring vital signs (body temperature, pulse rate, respiration rate)	1	2	3	4	5
7. Monitoring heart rhythm	1	2	3	4	5
8. Monitoring blood pressure	1	2	3	4	5
9. Measuring blood sugar levels	1	2	3	4	5
10. Detecting changes in skin responses (temperature, sweat secretion)	1	2	3	4	5
11. Administer oral medications	1	2	3	4	5
12. Administer intravenous (IV) medications	1	2	3	4	5
13. Performing nasal or oral swabs	1	2	3	4	5
14. Drawing blood for testing	1	2	3	4	5
15. Electrocardiogram	1	2	3	4	5

16. Behaviour change counselling or coaching	1	2	3	4	5
17. Home cleaning and sanitation	1	2	3	4	5
18. Providing companionship	1	2	3	4	5
19. Assisting with mobility (walking, sitting, moving arms/legs)	1	2	3	4	5
20. Assisting with eating, bathing, toileting, dressing	1	2	3	4	5
21. Recording/reviewing daily agenda	1	2	3	4	5
22. Cognitive training (recall games, puzzles)	1	2	3	4	5
23. Entertainment	1	2	3	4	5
24. Faith/ Spiritual activities	1	2	3	4	5
25. Symptom screening and triage	1	2	3	4	5

Other capabilities that would be helpful: ____

____

____

____

What do you see as benefits of, and important considerations for using a social robot that can actively monitor your health?

____

____

____

____

What could motivate you to purchase a social robot that can support your/your loved one's health?

____

____

____

____

If you can have a social robot or smart system at home to support, you for your /your loved one's health and health monitoring at home and what type of data would you allow to be gathered? (You can choose more than one option)

- ☐ My voice, regardless of whether I am interacting with the intelligent agent or not.
- ☐ My video, regardless of whether I am interacting with the intelligent agent or not.
- ☐ My voice, only when I am interacting with the intelligent agent.
- ☐ My video, only when I am interacting with the intelligent agent.
- ☐ Text conversations with you
- ☐ Physiological data (e.g., heart rate, blood pressure)
- ☐ Physical activity (e.g., via pedometer)
- ☐ Medical information (e.g., allergies, medical conditions, health provider's contact information)

Circle a number to indicate how comfortable/uncomfortable would you be with a social robot monitoring and tracking health data using each of the methods below (on a scale of 1-10):

Using wearable sensors

Highly uncomfortable 1--2---3---4---5---6---7---8---9---10 Highly comfortable

Using a smart home

Highly uncomfortable 1--2---3---4---5---6---7---8---9---10 Highly comfortable

Using other external devices/sensors

Highly uncomfortable 1--2---3---4---5---6---7---8---9---10 Highly comfortable

Study ID: ____________________
Date: _______________________

Select who would you allow to access the data gathered by a social robot or intelligent system. (You can choose more than one option)

I would share my data with:

- ☐ Anyone.
- ☐ Friends.
- ☐ Family members.
- ☐ My significant other.
- ☐ My family doctor.
- ☐ My specialist.
- ☐ No one. Data should be absolutely private and only be used to provide me or my loved one a report (which we can share with someone later).

Supplement 3

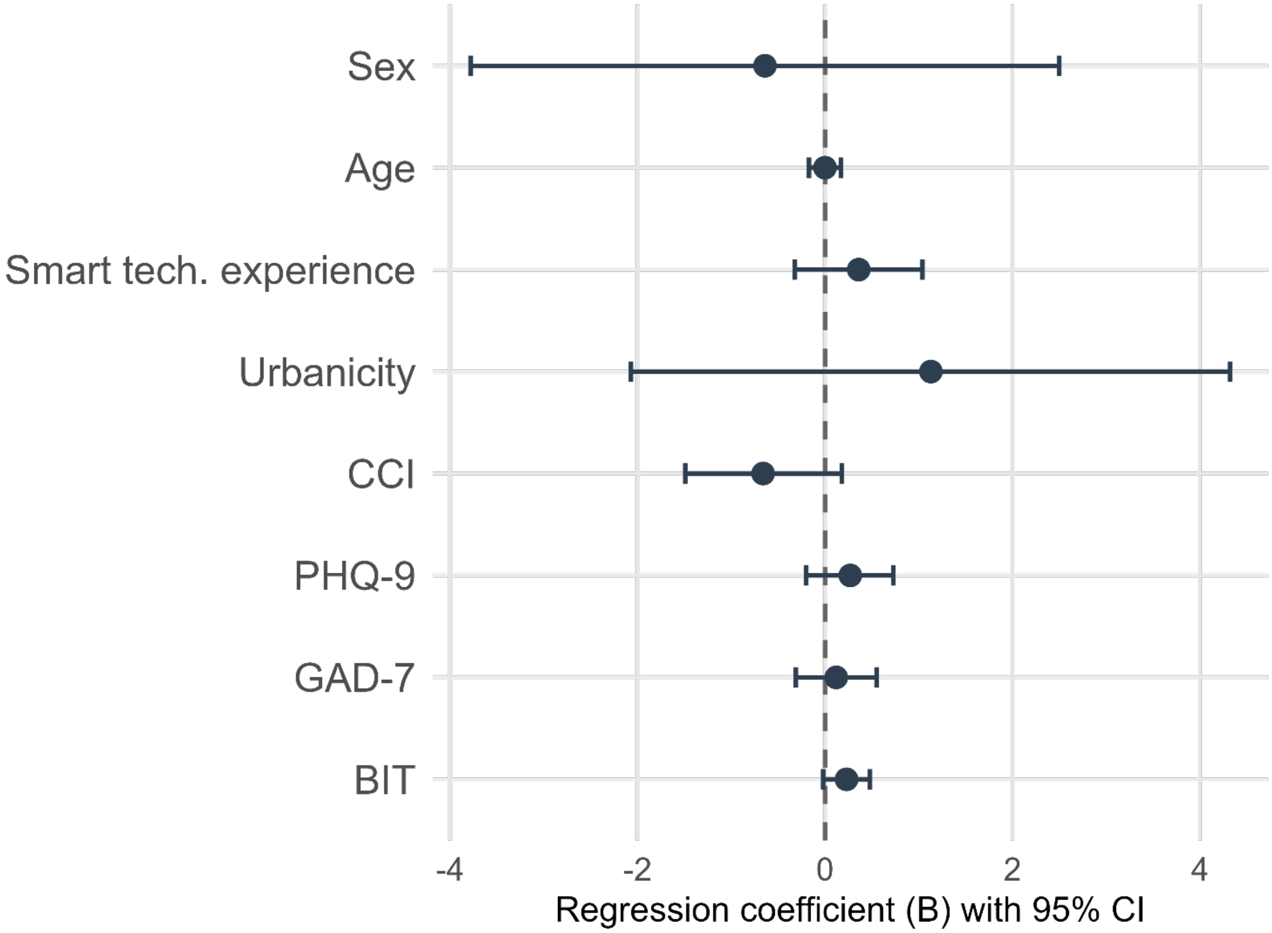

Figure S3. Forest plot of regression coefficients (B) and 95% CI for predictors of UTAUT performance expectancy. Sex: 0 = male, 1 = female; Smart tech. experience = number of devices in use; Urbanicity: 0 = non-urban, 1 = urban; CCI = Charlson Comorbidity Index; PHQ-9 = Patient Health Questionnaire-9; GAD-7 = Generalized Anxiety Disorder-7; BIT = Brief Inventory of Thriving.

Supplement 4

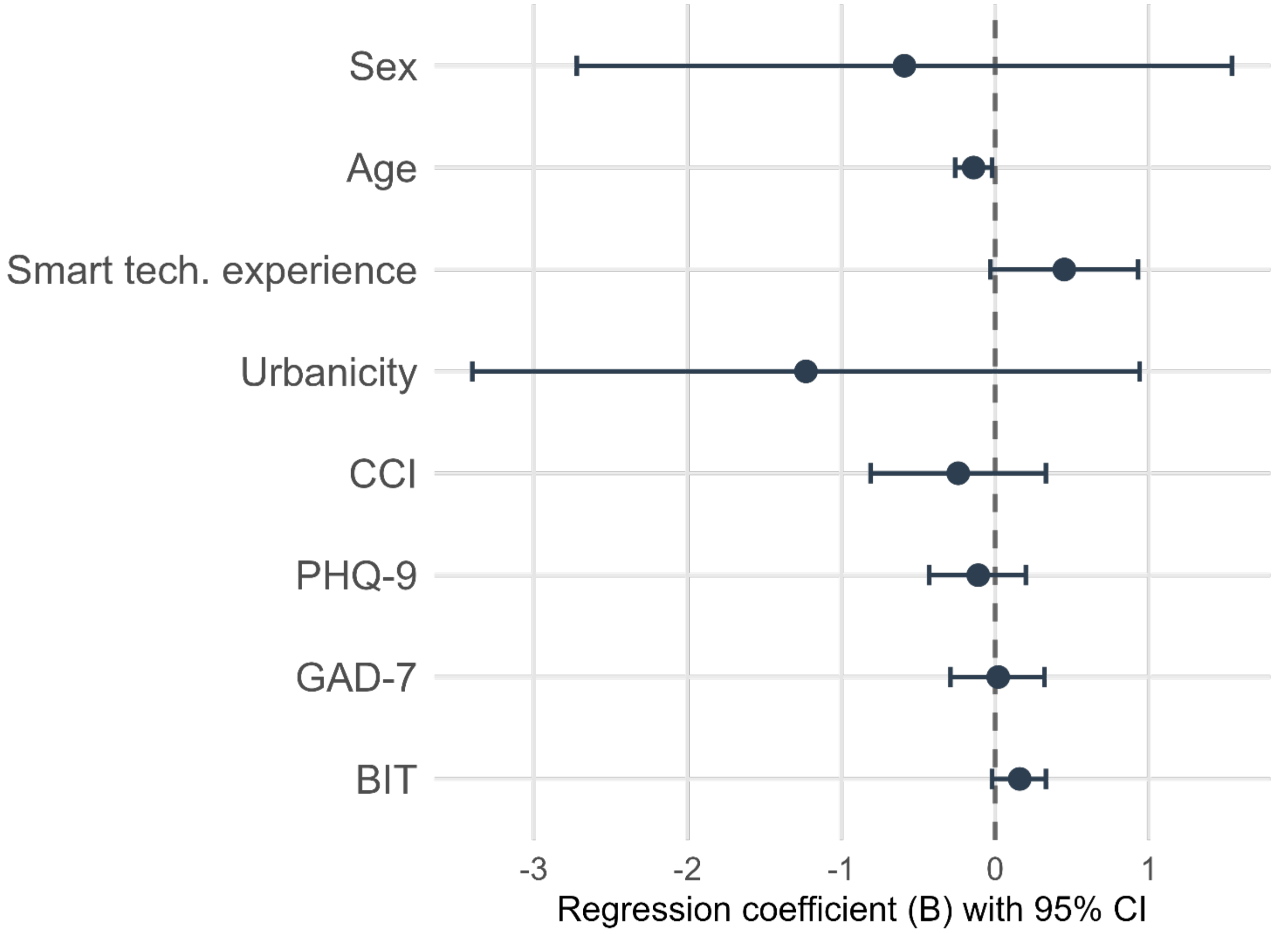

Figure S4. Forest plot of regression coefficients (B) and 95% CI for predictors of UTAUT effort expectancy. Sex: 0 = male, 1 = female; Smart tech. experience = number of devices in use; Urbanicity: 0 = non-urban, 1 = urban; CCI = Charlson Comorbidity Index; PHQ-9 = Patient Health Questionnaire-9; GAD-7 = Generalized Anxiety Disorder-7; BIT = Brief Inventory of Thriving.

Supplement 5

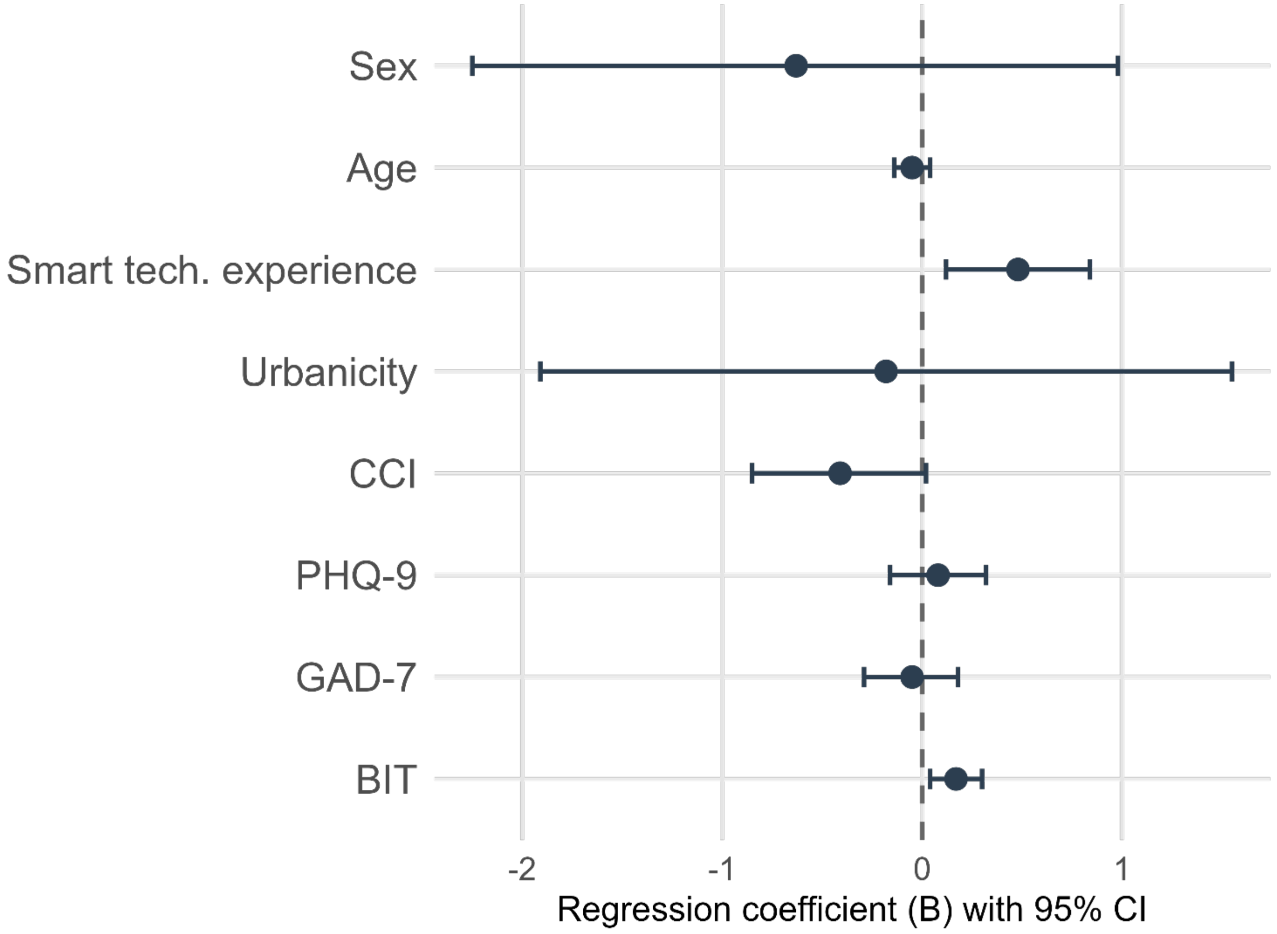

Figure S5. Forest plot of regression coefficients (B) and 95% CI for predictors of UTAUT facilitating conditions. Sex: 0 = male, 1 = female; Smart tech. experience = number of devices in use; Urbanicity: 0 = non-urban, 1 = urban; CCI = Charlson Comorbidity Index; PHQ-9 = Patient Health Questionnaire-9; GAD-7 = Generalized Anxiety Disorder-7; BIT = Brief Inventory of Thriving.

Supplement 6

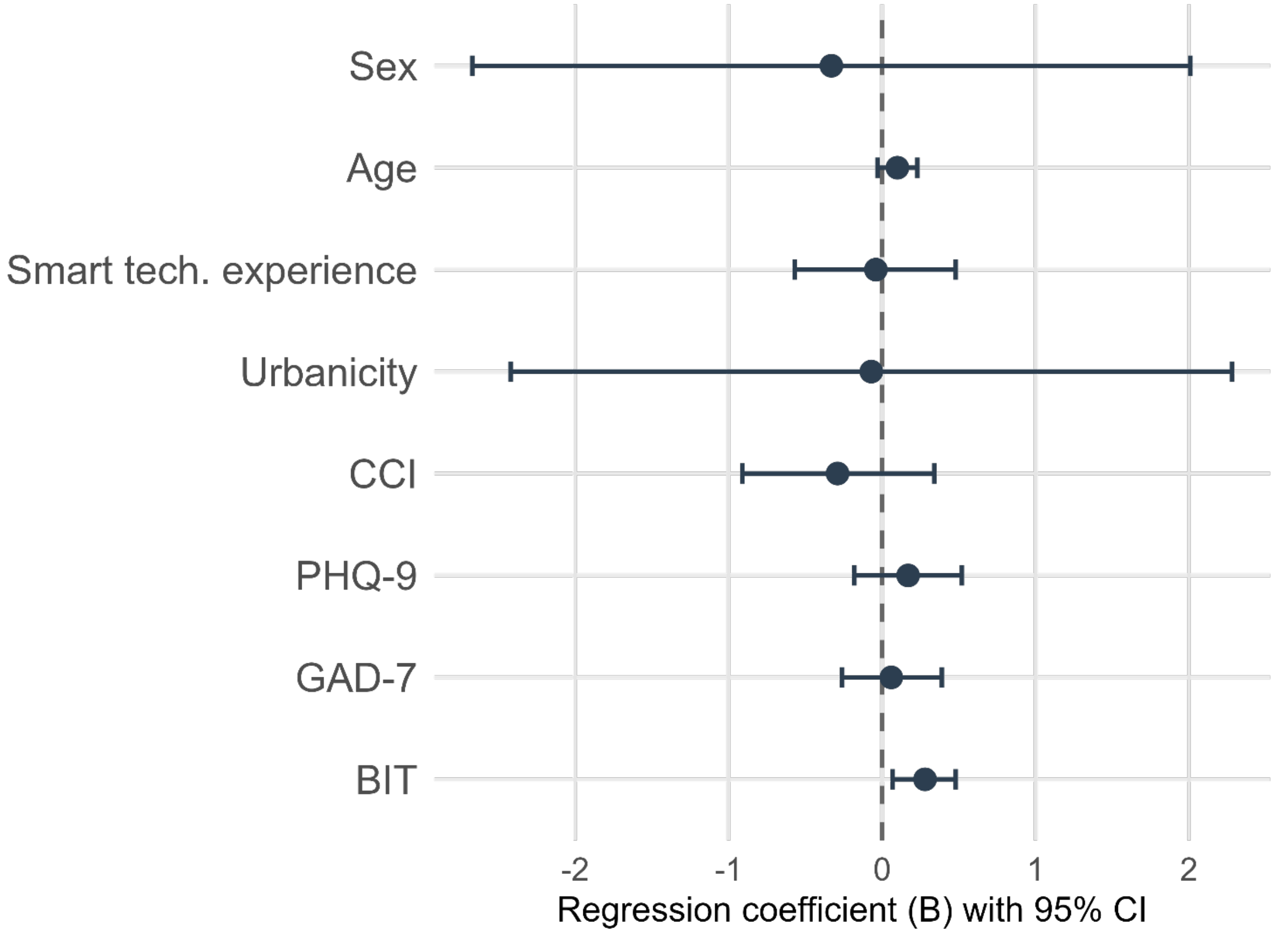

Figure S6. Forest plot of regression coefficients (B) and 95% CI for predictors of UTAUT social influences. Sex: 0 = male, 1 = female; Smart tech. experience = number of devices in use; Urbanicity: 0 = non-urban, 1 = urban; CCI = Charlson Comorbidity Index; PHQ-9 = Patient Health Questionnaire-9; GAD-7 = Generalized Anxiety Disorder-7; BIT = Brief Inventory of Thriving.

Supplement 7. Correlation Matrix of individual BIT items and 25 rated social robot capabilities and features

		1. Scheduling appointments	2. Facilitating a telemedicine interview	3. Reminders to take medication, exercise, or eat	4. Monitoring changes in mood	5. Detecting falls	6. Monitoring vital signs (body temperature, pulse rate, respiration rate)	7. Monitoring heart rhythm	8. Monitoring blood pressure	9. Measuring blood sugar levels	10. Detecting changes in skin responses (temperature, sweat secretion)
1. My life has a clear sense of purpose	Pearson Correlation	0.124	0.111	0.020	-0.107	0.061	0.022	-0.002	-0.040	-0.071	0.025
	Sig. (2-tailed)	0.233	0.286	0.849	0.302	0.559	0.832	0.986	0.700	0.494	0.813
	N	95	95	95	96	95	96	95	95	94	96
2. I am optimistic about my future	Pearson Correlation	-0.025	-0.073	-0.037	-0.194	-0.007	-0.044	-0.071	-0.119	-0.099	-0.060
	Sig. (2-tailed)	0.811	0.482	0.720	0.058	0.944	0.668	0.499	0.251	0.345	0.561
	N	95	95	95	96	95	96	95	95	94	96
3. My life is going well	Pearson Correlation	0.047	0.031	0.085	-0.080	0.054	0.023	0.015	-0.014	0.003	-0.034
	Sig. (2-tailed)	0.654	0.763	0.413	0.442	0.604	0.822	0.884	0.891	0.976	0.743
	N	95	95	95	96	95	96	95	95	94	96
4. I feel good most of the time	Pearson Correlation	0.090	0.040	0.088	-0.116	0.095	0.108	0.061	0.020	0.067	0.033
	Sig. (2-tailed)	0.385	0.701	0.399	0.263	0.359	0.295	0.556	0.851	0.520	0.751
	N	95	95	95	96	95	96	95	95	94	96
5. What I do in life is valuable and worthwhile	Pearson Correlation	0.134	0.099	0.096	-0.046	0.124	0.083	0.069	0.009	-0.003	0.088
	Sig. (2-tailed)	0.195	0.340	0.355	0.660	0.233	0.423	0.510	0.927	0.977	0.395
	N	95	95	95	96	95	96	95	95	94	96
6. I can succeed if I put my mind to it	Pearson Correlation	0.039	0.052	0.171	-0.125	0.001	0.111	0.054	0.044	-0.046	-0.078
	Sig. (2-tailed)	0.710	0.618	0.097	0.226	0.993	0.281	0.604	0.674	0.662	0.454
	N	95	95	95	96	95	96	95	95	94	96
7. I am achieving most of my goals	Pearson Correlation	0.130	0.118	0.127	-0.096	0.100	0.109	0.093	0.070	-0.013	0.009
	Sig. (2-tailed)	0.210	0.256	0.219	0.354	0.337	0.293	0.372	0.504	0.902	0.928
	N	95	95	95	96	95	96	95	95	94	96
8. In most activities I do, I feel energized	Pearson Correlation	0.132	0.115	0.137	0.012	0.090	0.116	0.045	0.042	0.040	0.041
	Sig. (2-tailed)	0.204	0.267	0.187	0.910	0.385	0.263	0.669	0.690	0.705	0.691
	N	95	95	95	96	95	96	95	95	94	96

9. There are people who appreciate me as a person	Pearson Correlation	0.037	0.132	0.148	0.014	0.043	0.014	0.008	-0.012	0.012	0.089
	Sig. (2-tailed)	0.724	0.203	0.152	0.891	0.683	0.893	0.938	0.910	0.912	0.391
	N	95	95	95	96	95	96	95	95	94	96
10. I feel a sense of belonging in my community	Pearson Correlation	0.111	0.111	0.141	0.065	0.209*	0.071	0.080	0.038	0.025	0.138
	Sig. (2-tailed)	0.285	0.285	0.174	0.530	**0.041**	0.491	0.443	0.718	0.811	0.180
	N	95	95	95	96	95	96	95	95	94	96
**BIT SUM Score**	Pearson Correlation	0.110	0.093	0.122	-0.091	0.102	0.082	0.047	0.005	-0.010	0.031
	Sig. (2-tailed)	0.289	0.368	0.241	0.381	0.325	0.428	0.654	0.960	0.926	0.763
	N	95	95	95	96	95	96	95	95	94	96

		11. Administer oral medications	12. Administer intravenous (IV) medications	13. Performing nasal or oral swabs	14. Drawing blood for testing	15. Electrocardiogram	16. Behavior change counselling or coaching	17. Home cleaning and sanitation	18. Providing companionship	19. Assisting with mobility (walking, sitting, moving arms/legs)	20. Assisting with eating, bathing, toileting, dressing
1. My life has a clear sense of purpose	Pearson Correlation	0.099	0.040	0.058	0.104	0.045	-0.024	0.072	-0.175	-0.110	-0.030
	Sig. (2-tailed)	0.339	0.700	0.577	0.315	0.669	0.817	0.487	0.088	0.290	0.777
	N	95	95	96	95	94	94	96	96	94	95
2. I am optimistic about my future	Pearson Correlation	-0.126	0.001	-0.019	-0.046	-0.081	-0.034	-0.055	-0.071	-0.019	-0.041
	Sig. (2-tailed)	0.228	0.989	0.852	0.663	0.442	0.743	0.594	0.493	0.856	0.694
	N	95	95	96	95	94	94	96	96	94	95
3. My life is going well	Pearson Correlation	0.049	0.054	-0.007	0.070	-0.001	0.008	0.020	-0.065	0.055	-0.013
	Sig. (2-tailed)	0.635	0.602	0.946	0.501	0.991	0.937	0.849	0.532	0.600	0.902
	N	95	95	96	95	94	94	96	96	94	95
4. I feel good most of the time	Pearson Correlation	0.060	0.053	0.002	0.114	0.086	0.042	0.021	-0.033	0.020	-0.028
	Sig. (2-tailed)	0.562	0.609	0.982	0.273	0.412	0.687	0.841	0.747	0.845	0.791
	N	95	95	96	95	94	94	96	96	94	95
5. What I do in life is	Pearson Correlation	0.094	0.069	0.067	0.086	0.199	0.014	0.135	-0.046	0.093	0.108

valuable and	Sig. (2-tailed)	0.366	0.507	0.515	0.408	0.054	0.897	0.190	0.654	0.373	0.300
worthwhile	N	95	95	96	95	94	94	96	96	94	95
6. I can succeed if I put my mind to it	Pearson Correlation	0.107	-0.033	-0.054	0.053	0.129	-0.078	0.221*	-0.113	-0.006	0.014
	Sig. (2-tailed)	0.304	0.750	0.605	0.611	0.217	0.455	**0.030**	0.275	0.955	0.894
	N	95	95	96	95	94	94	96	96	94	95
7. I am achieving most of my goals	Pearson Correlation	0.078	0.021	-0.050	0.073	0.132	0.001	0.213*	-0.124	0.027	0.012
	Sig. (2-tailed)	0.451	0.841	0.629	0.484	0.204	0.996	**0.037**	0.230	0.793	0.905
	N	95	95	96	95	94	94	96	96	94	95
8. In most activities I do, I feel energized	Pearson Correlation	0.075	0.054	0.012	0.085	-0.050	0.070	0.116	0.063	0.029	0.001
	Sig. (2-tailed)	0.472	0.604	0.910	0.415	0.632	0.504	0.262	0.541	0.780	0.991
	N	95	95	96	95	94	94	96	96	94	95
9. There are people who appreciate me as a person	Pearson Correlation	0.118	0.055	0.129	0.044	0.126	0.044	0.246*	0.032	0.073	0.108
	Sig. (2-tailed)	0.254	0.598	0.211	0.672	0.228	0.678	**0.015**	0.756	0.483	0.299
	N	95	95	96	95	94	94	96	96	94	95
10. I feel a sense of belonging in my community	Pearson Correlation	0.209*	0.117	0.187	0.158	0.156	0.148	0.259*	0.059	0.106	0.075
	Sig. (2-tailed)	**0.042**	0.261	0.068	0.127	0.134	0.154	**0.011**	0.567	0.311	0.470
	N	95	95	96	95	94	94	96	96	94	95
**BIT SUM Score**	Pearson Correlation	0.095	0.057	0.037	0.098	0.088	0.026	0.151	-0.063	0.032	0.021
	Sig. (2-tailed)	0.363	0.587	0.722	0.347	0.398	0.804	0.142	0.543	0.760	0.841
	N	95	95	96	95	94	94	96	96	94	95

		21. Recording/reviewing daily agenda	22. Cognitive training (recall games, puzzles)	23. Entertainment	24. Faith/spiritual activities	25. Symptom screening and triage
1. My life has a clear sense of purpose	Pearson Correlation	0.046	-0.048	0.125	0.058	0.054
	Sig. (2-tailed)	0.655	0.644	0.227	0.586	0.611
	N	96	96	95	92	93
2. I am optimistic	Pearson Correlation	0.051	0.021	0.153	0.085	0.016

about my future	Sig. (2-tailed)	0.622	0.841	0.139	0.424	0.882
	N	96	96	95	92	93
3. My life is going well	Pearson Correlation	0.052	0.076	0.144	0.068	0.047
	Sig. (2-tailed)	0.615	0.464	0.163	0.521	0.655
	N	96	96	95	92	93
4. I feel good most of the time	Pearson Correlation	0.126	0.064	0.167	0.054	0.043
	Sig. (2-tailed)	0.223	0.539	0.107	0.611	0.683
	N	96	96	95	92	93
5. What I do in life is valuable and worthwhile	Pearson Correlation	0.155	0.062	0.150	0.183	0.026
	Sig. (2-tailed)	0.132	0.549	0.147	0.081	0.804
	N	96	96	95	92	93
6. I can succeed if I put my mind to it	Pearson Correlation	0.163	0.079	0.104	0.062	-0.006
	Sig. (2-tailed)	0.113	0.444	0.319	0.557	0.955
	N	96	96	95	92	93
7. I am achieving most of my goals	Pearson Correlation	0.143	0.088	0.185	0.115	0.025
	Sig. (2-tailed)	0.164	0.397	0.073	0.277	0.816
	N	96	96	95	92	93
8. In most activities I do, I feel energized	Pearson Correlation	0.122	0.144	0.209^{*}	0.190	-0.027
	Sig. (2-tailed)	0.237	0.162	**0.042**	0.069	0.794
	N	96	96	95	92	93
9. There are people who appreciate me as a person	Pearson Correlation	0.263^{**}	0.119	0.219^{*}	0.164	0.093
	Sig. (2-tailed)	**0.009**	0.251	**0.033**	0.118	0.378
	N	96	96	95	92	93
10. I feel a sense of belonging in my community	Pearson Correlation	0.233^{*}	0.139	0.228^{*}	0.123	0.192
	Sig. (2-tailed)	**0.022**	0.177	**0.026**	0.244	0.065
	N	96	96	95	92	93
**BIT SUM Score**	Pearson Correlation	0.168	0.094	0.216^{*}	0.142	0.056
	Sig. (2-tailed)	0.102	0.361	**0.035**	0.178	0.594
	N	96	96	95	92	93

Note: Sample sizes in Supplement 7 (range: 94–96) reflect pairwise deletion for variables not included in the multiple imputation model, whereas the main text analyses are based on the multiply imputed dataset (N = 101). ****p* < 0.001, ***p* < 0.01, **p* < 0.05.
