# Supplementary figures and images for: Positive Psychological Well-Being and Determinants of Social Robot Acceptability Among Patients With Heart Failure: Cross-Sectional Questionnaire Study
Source: JMIR Cardio. 2026 Jun 2;10:e83163. doi: 10.2196/83163 (PMC13229465; doi:10.2196/83163)

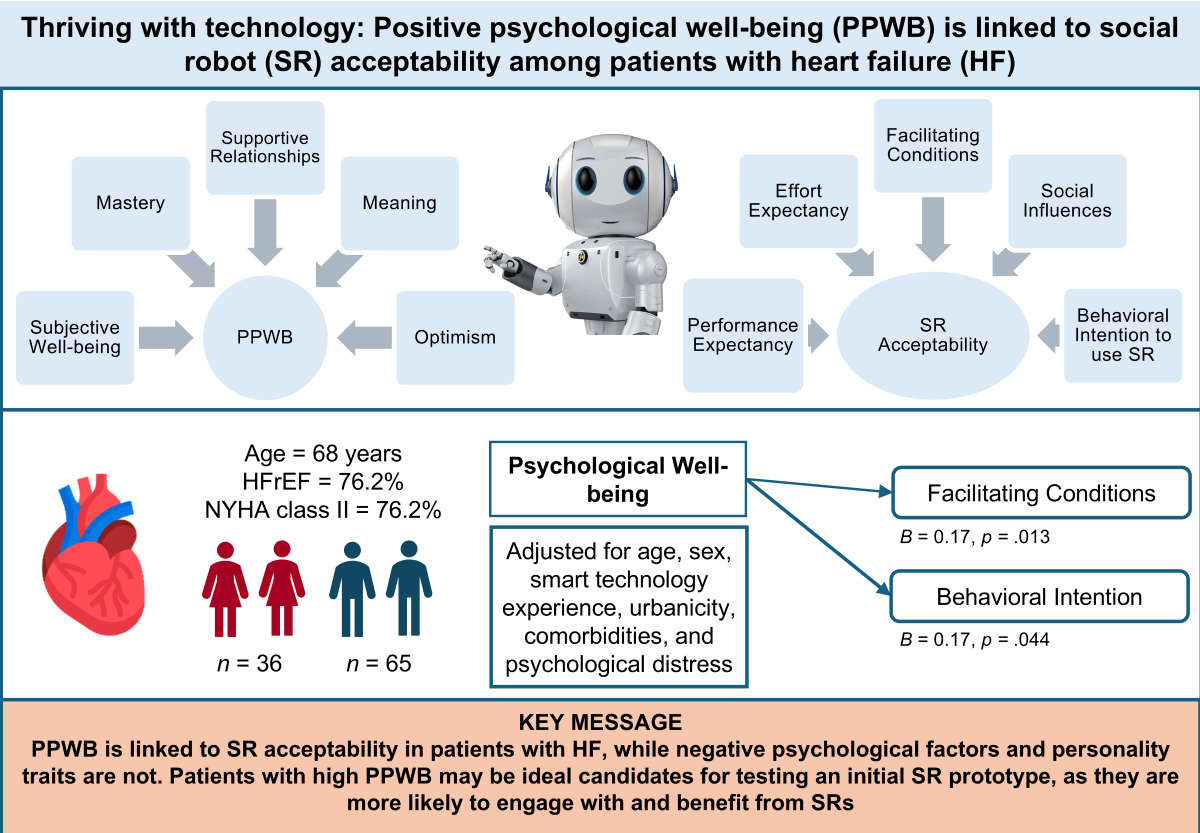

Supplement: Multimedia Appendix 2 [file cardio-v10-e83163-s002.png]
